# Association between personality types and low anterior resection syndrome in rectal cancer patients following surgery

**DOI:** 10.1002/cam4.7022

**Published:** 2024-02-24

**Authors:** Ting‐Yu Chiang, Yu‐Jen Hsu, Yih‐Jong Chern, Chun‐Kai Liao, Wen‐Sy Tsai, Pao‐Shiu Hsieh, Hung‐Chih Hsu, Yu‐Fen Lin, Hsiu‐Lan Lee, Jeng‐Fu You

**Affiliations:** ^1^ Department of Nursing, Chang Gung Medical Foundation Linkou Chang Gung Memorial Hospital Taoyuan Taiwan; ^2^ Division of Colon and Rectal Surgery, Department of Surgery, Chang Gung Memorial Hospital at Linkou, College of Medicine Chang Gung University Taoyuan Taiwan; ^3^ Division of Hematology‐Oncology, Chang Gung Memorial Hospital at Linkou, College of Medicine Chang Gung University Taoyuan Taiwan

**Keywords:** LAR syndrome, personality, rectal cancer, Type A, Type D

## Abstract

**Purpose:**

Low anterior resection syndrome (LARS) has had many impacts on the lives of patients and substantial differences in emotional and social functions. The aim of this study was to investigate the correlation analysis of different personality traits in rectal cancer patients with LARS after undergoing curative surgery.

**Methods:**

This study was designed as a prospective cohort study. The inclusion criteria included (1) participants diagnosed with rectal cancer who underwent surgical resection of malignant tumors and (2) ECOG 0–1. The primary outcome was the correlation between different personality traits and low anterior resection syndrome in rectal cancer patients after radical surgery. Low anterior resection syndrome incidence rates were estimated by questionnaires and personality groups by the Type A and Type D Scale‐14 Personality Inventory.

**Results:**

For all 161 participants in this study, the presence of a tumor at the lower anal verge and the receipt of neoadjuvant CCRT had a statistically significant positive correlation with the LARS score at 1 month, 6 months, and 1 year (Pearson correlation coefficient = −0.283, −0.374, and − 0.205, respectively), with a *p* value of less than 0.05. Personalities with Type A, Type D, and Type D‐SI scores had a statistically significant positive correlation with LARS score at 1 month (Pearson correlation coefficient = 0.172, 0.162, and 0,164, *p* value = 0.03, 0.04, and 0.04).

**Conclusion:**

Type A and Type D personalities are highly linked to LARS. Personalized support approaches can ultimately assist rectal cancer patients in overcoming difficulties after surgery and recovery and enhance their functional outcomes.

## 1 INTRODUCTION

Low anterior resection syndrome (LARS) is a common complication experienced by patients undergoing rectal cancer surgery. The incidence is approximately 19.6%–90%, particularly for those who receive low anterior resection (LAR) surgery.^1^ LARS is a cluster of symptoms that can include fecal incontinence, urgency, and incomplete evacuation, and it can significantly impact a patient's quality of life.^2^, ^3^ In addition to surgery, radiation therapy, chemotherapy, and targeted therapy, neoadjuvant concurrent chemoradiotherapy (CCRT) is a standard treatment for locally advanced rectal cancer and has been shown to increase survival rates.^4^ However, surgery involving total mesorectal excision and neoadjuvant CCRT may also increase the risk of LARS in patients undergoing subsequent LAR surgery.^5^, ^6^ The occurrence of LARS has had considerable consequences on the patient's life, including frequent defecation, reluctance to eat for fear of increasing the frequency of defecation, poor nighttime sleep due to frequent defecation, and hesitation in leaving the house.^7^, ^8^ Studies have also found notable variations in mental and social functions in colorectal cancer patients, who experience more severe low anterior resection syndrome following surgery.^9^, ^10^ Consequently, health care providers need to identify the effect of LARS on mental and social functioning and physical symptoms to provide comprehensive care and support for colorectal cancer patients after surgery.

Diagnosing and treating cancer is a long and arduous challenge and a stressful situation for cancer survivors, who must contend with adverse reactions to therapy and the fear of disease recurrence.^11^ Several studies have found that different personality traits can affect individuals' coping behaviors after illness or treatment.^12^ People with a tendency toward depression and poor social interaction are more likely to experience postoperative complications. Colorectal cancer patients with anxiety or depression personality tendencies also have poorer postoperative health and survival rates.^13^ Personality types, such as Type A and Type B,^14^ can affect how cancer patients cope with their disease.^15^ Type A personalities are typically characterized as competitive, ambitious, and driven, while Type B personalities are more relaxed, laid‐back, and easygoing.^16^ Research has shown that Type A personalities may have a harder time coping with stress, including stress related to illness, than Type B personalities. They may be more likely to experience negative emotions such as anxiety and depression and may be less likely to use positive coping strategies, such as seeking social support and engaging in problem solving.^17^, ^18^, ^19^

The Type D personality is characterized by a tendency to experience negative emotions such as anxiety, depression, and social inhibition.^20^ According to research, Type D personality is linked with lower adherence among patients with chronic diseases, such as cancer.^21^, ^22^ They may have a more difficult time coping with the emotional and social challenges of the condition, and such difficulty has been connected to a higher risk of LARS in patients with rectal cancer.^23^ This provides support for the hypothesis that psychological characteristics are crucial in the emergence of LARS.^24^

In this research, we aim to investigate the relationship between varied personality traits and low anterior resection syndrome (LARS) in rectal cancer patients who have undergone radical surgery. The study is intended to improve patients' comprehension of the rationale behind disease treatment and psychological recovery following surgery, improve their ability and interest in self‐care, and ultimately enhance their postoperative quality of life. And also highlights the necessity of combining personal characteristics with medical treatment to obtain patient consensus on treatment options, a prime goal of contemporary precision medicine.

## 2 MATERIALS AND METHODS

### 2.1 Design and participants

This study is designed as a prospective cohort study: a single‐center study in the outpatient clinic for colorectal surgery at Chang Gung Memorial Hospital, Linkou Branch, from September 2020 to April 2022. Subject recruitment is based on a strategy of convenience sampling of participants. Throughout our investigation, participants were instructed to approach the task with emotional neutrality and tranquility, ensuring that their responses reflected a genuine cognitive domain. Participants were encouraged to postpone the assessment if they experienced excessive levels of negative emotions or anxiety to obtain a more accurate representation of their typical character and achieve emotional stability. Participants were also strongly encouraged to prioritize mental concentration and complete the assessment when not under time constraints to facilitate deliberate and mindful responses.

The study investigated patients who fulfilled the following inclusion criteria: (1) diagnosis of rectal cancer; (2) surgical excision of primary malignant tumors; (3) Eastern Cooperative Oncology Group (ECOG) performance status of 0–1; and (4) signed informed consent. Patients excluded from the study were (1) those who were either acutely or chronically mentally ill, exhibited cognitive impairment, or were unable to cooperate and (2) patients who had undergone local excision or abdominal perineal resection (APR).

### 2.2 Sample size

The sample size estimation was based on 16 variables, including patient characteristics, treatment status, low anterior resection syndrome score, Type A personality trait, Type D personality trait, and colorectal cancer functional assessment of quality of life. Gpower3.1.2 software was used to estimate the sample size, with *α* = 0.05, power = 0.8, and effect size = 0.5. The sample size was 160. To reduce the error caused by invalid questionnaires and missing values, 10% of the sample size was added, resulting in 176. In addition, five subjects were included in the pilot study, totaling 181 subjects.

### 2.3 Variables and measurement outcomes

Baseline characteristics included age, sex, body mass index (BMI), neoadjuvant CCRT, primary tumor location from the anal verge, preoperative clinical stage, and postoperative pathology stage. These variables were utilized to better understand the study population and explore potential differences or similarities within subgroups of participants. The survival outcome result was taken from the Cancer Registry Database of a medical center in northern Taiwan.

### 2.4 Low anterior resection syndrome (LARS) score

The functional outcome was assessed with the LARS score^25^ at 1 month, 6 months, and 1 year. LARS scoring evaluates flatus incontinence (0–7 points), liquid stool incontinence (0–3 points), evacuation frequency (0–5 points), re‐evacuation (0–11 points), and evacuation urgency (0–16 points). A score of 0–20 points indicates no LARS, 21–29 points indicates minor LARS, and 30–42 points indicates major LARS.

### 2.5 Types of personality assessment

The Type A Personality Inventory Bortner Scale^26^, ^27^ was developed jointly by Professor Bortner of Human Development and Dr. Rosenman, a cardiologist, in the United States. This scale was designed to evaluate personality traits related to coronary heart disease (CHD) among patients and measures 14 rating scales, which, in extreme cases, are typical of Type A versus Type B personality. The reliability of this scale range from 0.53 to 0.68, implies a level of reliability that can be characterized as marginal.^28^ This implies that, on average, the items within the Bortner Scale of the Type A Personality Inventory exhibit correlation, indicating a reasonable degree of internal coherence in assessing the characteristics linked with Type A and Type B personalities. On a scale of 1–11, participants assess their agreement with each statement. A lower score implies that the statement does not match the participant's personality characteristics, whereas a higher score suggests that the statement does represent the participant's traits. This scoring system is used to determine the extent to which the characteristics of the participants correspond to the characteristics being evaluated. A total score ranging from 14 to 63 indicates extreme Type‐B personality; scores between 64 and 92 indicate moderate Type‐B personality; scores between 93 and 107 indicate moderate Type A personality; and scores between 108 and 154 indicate excessive Type A personality.

The Type D Scale‐14 Personality Inventory was designed by Dutch scholar Johan Denollet in 1995 to study the personality traits of coronary heart disease patients by the Type D personality theory.^29^, ^30^ Negative affectivity (NA) and social inhibition (SI) are two of the key personality traits assessed by this exam. Individuals with high levels of NA experience negative emotions such as anxiety, depression, and irritability and tend to dwell on negative thoughts and feelings. On the contrary, individuals with high levels of SI tend to avoid social situations and interactions, often due to fear of rejection or negative evaluation by others. The Type D Scale‐14 questionnaire consists of 14 items, with seven items each for NA and SI and a reliability of 0.86–0.88. Each question is scored on a scale of 0–4, and the total score ranges from 0 to 56. The higher the score, the greater the inclination toward Type D personality, which indicates a tendency to suppress self‐expression in social life and lower levels of hostile expression.

### 2.6 Statistical analyses

All analyses were conducted using SPSS Statistics v.26 (IBM Corp., Armonk, NY, USA). An *α* level of 0.05 was considered statistically significant, and all *p* values were two‐sided. The baseline characteristics of the LARS and personality group are presented as categorical variables, presented as frequencies and proportions, and were compared using the X ^2^ test. Continuous variables are expressed as the means and standard deviations and were analysed using Student's *t*‐test and one‐way ANOVA. The correlation was analysed using multivariate analysis by linear regression.

## 3 RESULTS

### 3.1 Demographic and clinical characteristics

This study involved enrolling 161 patients with rectal cancer who underwent radical resection and the demographic and characteristics in Table 1.

**TABLE 1:** Patient characteristics (*n* = 161).

	Total	%
Age, range (median ± SD)	30–84 (58.76 ± 9.71)	
≦50	34	21.1
51–70	111	68.9
>70	16	9.9
Sex		
M	101	62.7
F	60	37.3
BMI (kg/m^2^) mean ± SD	13.91–38.97 (24.28 ± 3.78)	
≦25	106	65.8
>25	55	34.2
Comorbidities		
DM	30	18.7
HTN	36	22.3
Others	32	19.8
Neoadjuvant CCRT		
No	113	70.2
Yes	48	29.8
Anal verge		
≦5	34	21.1
6 ~ 10	78	48.4
>10	49	30.4
Clinical stage		
1	35	21.7
2	30	18.6
3	71	44.1
4	25	15.5
Pathology stage		
0	3	1.9
1	42	26.1
2	31	19.3
3	60	37.3
4	25	15.5
Postoperative LARS score		
1 month, range (median ± SD)	0–41 (21.96 ± 13.20)	
No LARS	65	40.4
Minor LARS	33	20.5
Major LARS	63	39.1
6 months, range (median ± SD)	0–41 (13.35 ± 12.60)	
No LARS	104	64.6
Minor LARS	26	16.1
Major LARS	20	12.4
Missing	11	6.8
1 year, range (median ± SD)	0–41 (10.21 ± 10.85)	
No LARS	102	63.4
Minor LARS	15	9.3
Major LARS	9	5.6
Missing	35	21.7

Abbreviations: BMI, body mass index; CCRT, concurrent chemotherapy and radiotherapy; LARS score, low anterior resection syndrome score.

### 3.2 The personality type of the patient based on the Type A Scale score

The Type A Scale score ranges from 61 to 132 with a median of 94.87 and a standard deviation of 15.40. The table presents data on patients' personality types (Type A) and various variables, including age, sex, BMI (body mass index), comorbidities, clinical stage, pathology stage, and LARS (low anterior resection syndrome) scores at different time points (1 month, 6 months, and 1 year). At 1 month after surgery, the occurrence of major LARS was higher among individuals with moderate Type‐A personality (38.1%) and extreme Type‐A personality (31.7%) than among those with extreme Type‐B personality (1.6%) and moderate Type‐B personality (28.6%). Despite the higher occurrence of LARS among Type‐A personalities, the *p* value of 0.06 suggests that there may be a possible relationship between personality types and the development of LARS, but the evidence is not statistically significant (Table 2).

**TABLE 2:** Personality of Type A with variable.

		Extreme Type‐B	Moderate Type‐B	Moderate Type‐A	Extreme Type‐A	*p*
		*n* (%)	*n* (%)	*n* (%)	*n* (%)	
Range (median ± SD) 61–132 (94.87 ± 15.40)						
Age	≦50	0 (0.0)	13 (38.2)	11 (32.4)	10 (29.4)	0.83
	51–70	2 (1.8)	49 (44.1)	33 (29.7)	27 (24.3)	
	>70	0 (0.0)	8 (50.0)	6 (37.5)	2 (12.5)	
Sex	F	0 (0.0)	28 (46.7)	18 (30.0)	14 (23.3)	0.69
	M	2 (2.0)	42 (41.6)	32 (31.7)	25 (24.8)	
BMI	<25	2 (1.9)	49 (46.2)	32 (30.2)	23 (21.7)	0.48
	≧25	0 (0.0)	21 (38.2)	18 (32.7)	16 (29.1)	
Comorbidities	DM	1 (3.3)	11 (36.7)	12 (40)	6 (20)	0.41
	HTN	0 (0)	19 (52.8)	12 (33.3)	5 (13.9)	0.31
	Others	1 (3.1)	13 (40.6)	11 (34.4)	7 (21.9)	0.70
Clinical stage	1	1 (2.9)	11 (31.4)	11 (31.4)	12 (34.3)	0.76
	2	0 (0.0)	14 (46.7)	10 (33.3)	6 (20.0)	
	3	1 (1.4)	35 (49.3)	21 (29.6)	14 (19.7)	
	4	0 (0.0)	10 (40.0)	8 (32.0)	7 (28.0)	
Pathology stage	0	0 (0.0)	2 (66.7)	1 (33.3)	0 (0.0)	0.80
	1	1 (2.4)	20 (47.6)	12 (28.6)	9 (21.4)	
	2	0 (0.0)	13 (41.9)	7 (22.6)	11 (35.5)	
	3	1 (1.7)	25 (41.7)	23 (38.3)	11 (18.3)	
	4	0 (0.0)	10 (40.0)	7 (28.0)	8 (32.0)	
LARS 1 month	No LARS	0 (0.0)	37 (56.9)	17 (26.2)	11 (16.9)	0.06
	Minor LARS	1 (3.0)	15 (45.5)	9 (27.3)	8 (24.2)	
	Major LARS	1 (1.6)	18 (28.6)	24 (38.1)	20 (31.7)	
LARS 6 months	No LARS	2 (1.9)	48 (46.2)	30 (28.8)	24 (23.1)	0.89
	Minor LARS	0 (0.0)	10 (38.5)	9 (34.6)	7 (26.9)	
	Major LARS	0 (0.0)	8 (40.0)	8 (40.0)	4 (20.0)	
LARS 1 year	No LARS	1 (1.0)	47 (46.1)	34 (33.3)	20 (19.6)	0.75
	Minor LARS	1 (6.7)	6 (40.0)	5 (33.3)	3 (20.0)	
	Major LARS	0 (0.0)	5 (55.6)	3 (33.3)	1 (11.1)	

Abbreviations: BMI, body mass index; CCRT, concurrent chemotherapy and radiotherapy; DM, diabetes mellitus; HTN, hypertension; LARS score: low anterior resection syndrome score.

The correlation of personality with variables and measurement outcomes is shown in Table 3. This table provides information on the correlation between personality scores (Type A and Type D) and various variables and outcomes in the study population. Positive correlations indicate that higher personality scores are associated with higher values of the variable or measurement outcome, while negative correlations indicate an inverse relationship. *p* values provide information about the statistical significance of the correlations, with lower values indicating stronger evidence of a relationship.

**TABLE 3:** Correlation of personality with variables and measurement outcomes.

	Type A score		Type D score	
	Correlation	*p*	Correlation	*p*
Age	−0.20	0.01	−0.18	0.02
Sex	0.02	0.83	−0.03	0.71
BMI	0.20	0.01	−0.13	0.10
Anal verge	0.03	0.68	0.04	0.64
Clinical stage	−0.04	0.59	0.05	0.50
Pathology stage	0.10	0.21	0.03	0.69
LARS 1 month	0.18	0.02	0.16	0.04
LARS 6 month	0.09	0.26	0.15	0.07
LARS 1 year	0.05	0.61	0.09	0.32
Type A score			0.18	0.03
Type D score	0.18	0.03		
Type D‐NA	0.13	0.11		
Type D‐SI	0.22	0.01		

Abbreviations: BMI, body mass index; LARS, low anterior resection syndrome; Type D‐NA, negative affectivity; Type D‐SI, social inhibition.

Table 4 is the correlation between the variables including age, sex, BMI, anal verge, neoadjuvant CCRT, preoperative clinical stage, pathology stage, Type A score, Type D score, Type D‐NA score, and Type D‐SI score with LARS score at 1 month, 6 months, and 1 year after surgery. Age and sex had negative correlation with LARS score. But tumors at the lower anal verge and receiving neoadjuvant CCRT had a statistically significant positive correlation with the LARS score at 1 month, 6 months, and 1 year (Pearson correlation coefficient = −0.283, −0.374, and −0.205, respectively), with a *p* value of less than 0.05. This indicates that as the distance of the tumor from the anal verge decreases, the LARS score improves. Personality with Type A, Type D, and Type D‐SI scores had a statistically significant positive correlation with LARS score at 1 month (Pearson correlation coefficient = 0.172, 0.162, and 0,164, *p* value = 0.03, 0.04, and 0.04) (Table 4).

**TABLE 4:** Correlation of variables with LARS score at 1 month, 6 months, and 1 year (*N* = 161).

Variable	1 month			6 months			1 year		
	Pearson correlation	Mean ± SD	*p*	Pearson correlation	Mean ± SD	*p*	Pearson correlation	Mean ± SD	*p*
Age	−0.08	58.76 ± 9.71	0.32	0.02	58.76 ± 9.71	0.82	0.08	58.76 ± 9.71	0.39
Sex			0.41			0.13			0.21
BMI	0.04	24.27 ± 3.79	0.64	0.07	24.27 ± 3.79	0.43	−0.03	24.27 ± 3.79	0.77
Anal verge	−0.283**	9.20 ± 3.40	0.00**	−0.374**	9.20 ± 3.40	0.00**	−0.205*	9.20 ± 3.40	0.02*
Neoadjuvant CCRT			0.00**			0.00**			0.02*
Preop clinical Stage			0.77			0.11			0.19
Pathology Stage			0.20			0.65			0.46
Type A score	0.172*	94.87 ± 15.40	0.03*	0.08	94.87 ± 15.40	0.31	0.06	94.87 ± 15.40	0.55
Type D score	0.162*	16.72 ± 8.16	0.04*	0.15	16.72 ± 8.16	0.07	0.09	16.72 ± 8.16	0.32
Type D‐NA score	0.14	7.78 ± 5.83	0.08	0.15	7.78 ± 5.83	0.07	0.09	7.78 ± 5.83	0.32
Type D‐SI score	0.164*	8.94 ± 3.20	0.04*	0.11	8.94 ± 3.20	0.19	0.06	8.94 ± 3.20	0.48

Abbreviations: BMI, body mass index; neoadjuvant CCRT, concurrent chemotherapy with radiotherapy; LARS, low anterior resection syndrome; Type D‐NA, negative affectivity; Type D‐SI, social inhibition.

* *p* < 0.05.

** *p* < 0.01.

The results of multivariate analysis for the association between various clinical variables and LAR scores at 1 month, 6 months, and 1 year after surgery. The results suggest that at 1 month after surgery, the tumor at the lower anal verge and Type A score were significantly associated with the LAR score. At 6 months after surgery, only neoadjuvant CCRT was significantly (*p* value<0.001) associated with the LAR score. At 1 year after surgery, neoadjuvant CCRT was significantly (*p* value = 0.001) associated with the LAR score. The Type D score and Type D‐SI score were not found to be significantly associated with the LAR score at any time point. The VI values for all variables were below 4, indicating low‐to‐moderate levels of multicollinearity (Table 5).

**TABLE 5:** Association of clinical variables with LAR score in 1 month, 6 months, and 1 year by multivariate analysis (*N* = 161).

Variable	1 month			6 months		1 year			
	*𝞫*	*p*	VI	*p*	VI	*p*	VI	Adjusted R^B^	*F*
								0.07	2.76
Anal Verge	−0.27	0.05	1.53	0.00**	1.55	0.43	1.40		
Neo CCRT	6.44	0.07	1.52	0.11	1.54	0.01*	1.41		
Type A score	0.07	0.05	1.05	0.28	1.05	0.30	1.09		
Type D score	0.16	0.53	3.09	0.15	3.28	0.43	2.83		
Type D‐SI score	−0.10	0.56	3.15	0.73	3.34	0.84	2.88		

Abbreviations: Neo CCRT, neoadjuvant concurrent chemotherapy and radiotherapy; Type D‐SI, social inhibition.

* *p* < 0.05.

** *p* < 0.01.

**FIGURE 1:**
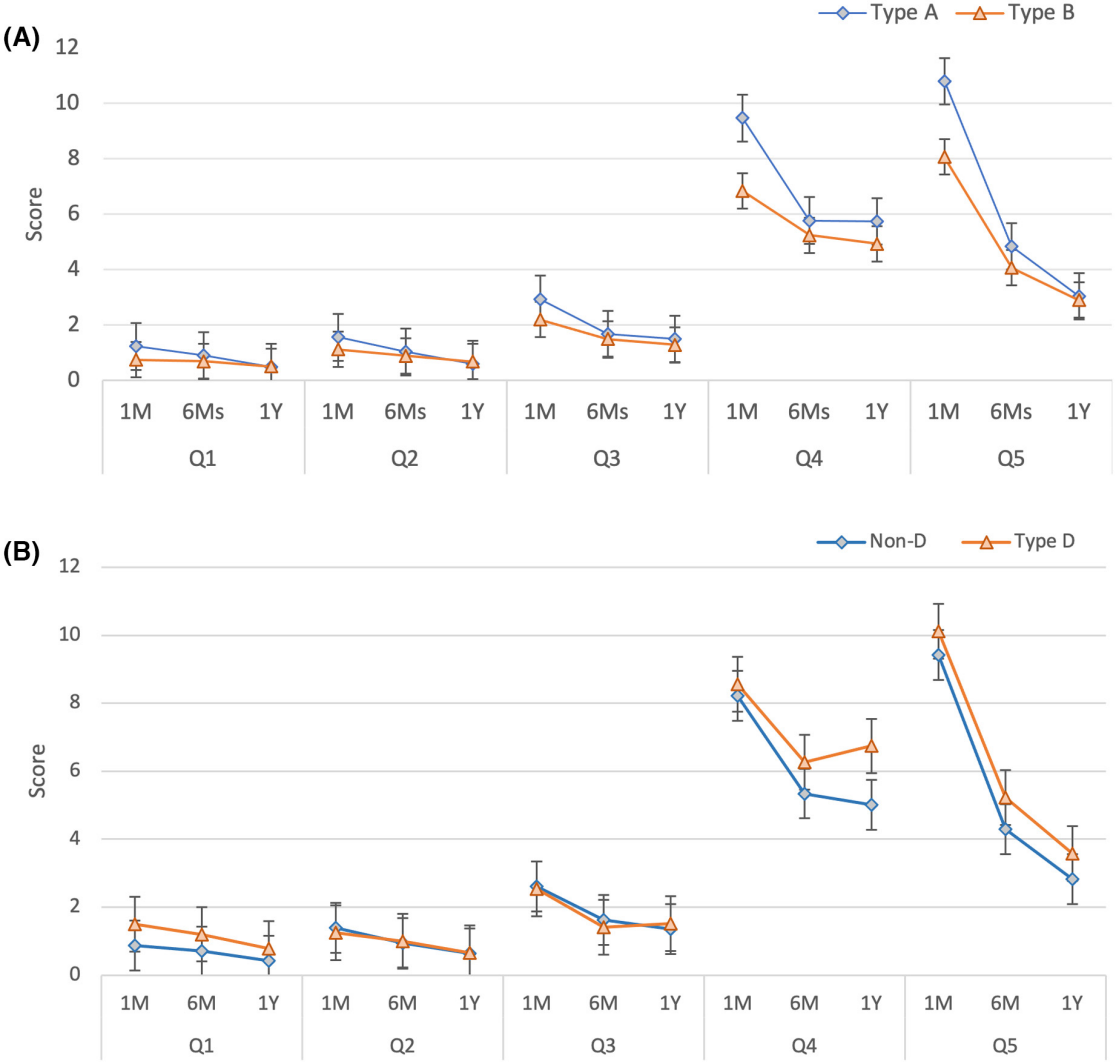
(A) LARS of personality with Type A and Type B after surgery by follow‐up at 1, 6 months, and 1 year; (B) LARS of personality with Type D and Non‐Type D after surgery by follow‐up at 1, 6 months, and 1 year; Q1, incontinence for flatus; Q2, incontinence for liquid stools; Q3, frequency; Q4, clustering; Q5, urgency; LARS, low anterior syndrome score; 1 M, 1 month; 6 M, 6 months; 1 Y, 1 year.

Figure 1A reveals the LARS of participants of Type A and Type B personality. The reported values were as follows: (1) compared with 1 month, the subscales of incontinence for flatus (mean [SD] score, 1.2 (2.3) vs. 0.7 (1.9); *p* = 0.001), incontinence (mean [SD] score, 1.6 (1.5) vs. 1.1 (1.5); *p* = 0.019), frequency (mean [SD] score, 2.9 (1.3) vs. 2.2 (1.6); *p* = 0.328), clustering (mean [SD] score, 9.5 (3.5) vs. 6.8 (5.2); *p* = <0.001), and urgency (mean [SD] score, 10.8 (6.7) vs. 8.1 (7.3); *p* = 0.008); (2) in the 6 months, the subscales of incontinence for flatus (mean [SD] score, 0.9 (2.1) vs. 0.7 (2.0); *p* = 0.264), incontinence (mean [SD] score, 1.0 (1.4) vs. 0.9 (1.4); *p* = 0.216), frequency (mean [SD] score, 1.7 (1.5) vs. 1.5 (1.5); *p* = 0.486), clustering (mean [SD] score, 5.8 (5.3) vs. 5.2 (5.3); *p* = 0.817), urgency (mean [SD] score, 4.8 (6.4) vs. 4.1 (6.3); *p* = 0.318); (3) in the 1 year, the subscales of incontinence for flatus (mean [SD] score, 0.5 (1.7) vs. 0.5 (1.7); *p* = 0.875), incontinence (mean [SD] score, 0.6 (1.2) vs. 0.7 (1.3); *p* = 0.468), frequency (mean [SD] score, 1.5 (1.5) vs. 1.3 (1.6); *p* = 0.353), clustering (mean [SD] score, 5.7 (5.2) vs. 4.9 (5.2); *p* = 0.886), and urgency (mean [SD] score, 3.0 (5.5) vs. 2.9 (5.6); *p* = 0.888).

The results showed that Type A personality was significantly associated with higher scores on several subscales of the LAR, indicating more severe bowel symptoms. Specifically, compared to those with Type B personality, those with Type A personality had higher scores on the subscales of flatus and incontinence at 1 month and higher scores on the incontinence, clustering, and urgency subscales at 1 month and 6 months. Additionally, those with Type A personality had significantly higher clustering scores at 1 month compared to those with Type B personality.

Figure 1B presents the LARS of the personalities in Type D and Non‐Type D groups. Compared with 1 month, the subscales of incontinence for flatus (mean [SD] score, 1.5 (2.7) vs. 0.9 (2.0); *p* = 0.005), incontinence (mean [SD] score, 1.2 (1.5) vs. 1.4 (1.5); *p* = 0.211), frequency (mean [SD] score, 2.5 (1.5) vs. 2.6 (1.5); *p* = 0.751), clustering (mean [SD] score, 8.6 (4.5) vs. 8.2 (4.6); *p* = 0.58), and urgency (mean [SD] score, 10.1 (6.9) vs. 9.4 (7.1); *p* = 0.311) were reported; in the 6 months, the subscales of incontinence for flatus (mean [SD] score, 1.2 (2.5) vs. 0.7 (1.9); *p* = 0.03), incontinence (mean [SD] score, 1.0 (1.4) vs. 1.0 (1.4); *p* = 0.74), frequency (mean [SD] score, 1.6 (1.6) vs. 1.0 (1.4); *p* = 0.08), clustering (mean [SD] score, 6.3 (5.3) vs. 5.3 (5.3); *p* = 0.22), and urgency (mean [SD] score, 5.2 (6.7) vs. 4.3 (6.3); *p* = 0.26) were reported; and in the 1 year, the subscales of incontinence for flatus (mean [SD] score, 0.8 (2.1) vs. 0.4 (1.5); *p* = 0.072), incontinence (mean [SD] score, 0.7 (1.3) vs. 0.6 (1.2); *p* = 0.851), frequency (mean [SD] score, 1.4 (1.6) vs. 0.7 (1.3); *p* = 0.017), clustering (mean [SD] score, 6.7 (5.1) vs. 5.0 (5.2); *p* = 0.061), and urgency (mean [SD] score, 3.6 (5.6) vs. 2.8 (5.5); *p* = 0.444) were reported.

In the study, compared to Non‐Type D participants, those with Type D personality showed significantly higher scores for incontinence for flatus and urgency at the 1‐month follow‐up but not at 6 months or 1 year. The frequency subscale score was also higher in Type D participants at the 1‐year follow‐up. Interestingly, while Type D participants had higher scores for incontinence for flatus and urgency at the 1‐month follow‐up, their scores decreased to be like those of Non‐Type D participants at 6 months and 1 year. This suggests that the effect of Type D personality on these subscales may be temporary and may not have a long‐term impact.

**FIGURE 2:**
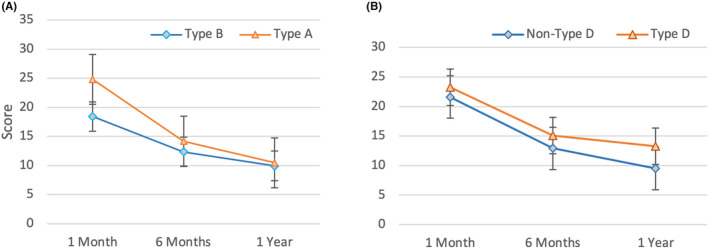
(A) Total LARS score of patients with Type A and Type B personality after surgery by follow‐up at 1, 6 months, and 1 year; (B) Total LARS score of patients with personality Type D and Non‐Type D after surgery by follow‐up at 1, 6 months, and 1 year; LARS, low anterior syndrome score; 1 M, 1 month; 6 M, 6 months; 1Y, 1 year.

Figure 2A presents the total LARS scores of the personalities classified in Type A and Type B groups. Compared with 1 month of the total scores (mean [SD] score, 24.8 (12.3) vs. 18.4 (13.5); *p* = 0.118), 6 months of the total scores (mean [SD] score, 14.2 (12.7) vs. 12.3 (12.4); *p* = 0.627), and 1 year of the total scores (mean [SD] score, 10.5 (10.3) vs. 9.9 (11.5); *p* = 0.596) were reported. The result was no significant difference in the total LARS scores between the Type A and Type B personality groups at 1 month, 6 months, and 1 year. This suggests that there is no significant association between personality type and bowel function in this population.

Figure 2B presents the total LARS scores of the personality with Type D and Non‐Type D groups. Compared with 1 month of the total scores (mean [SD] score, 23.3 (13.4) vs. 21.6 (13.2); *p* = 0.958), 6 months of the total scores (mean [SD] score, 1 15.1 (13.3) vs.; 12.9 (12.40); *p* = 0.65), and 1 year of the total scores (mean [SD] score, 13.3 (10.9) vs. 9.5 (10.8); *p* = 0.992) were reported. There were no significant differences in the total LARS scores between the Type D and Non‐Type D groups at any of the three time points (1 month, 6 months, and 1 year). This suggests that Type D personality was not associated with greater levels of fecal incontinence or bowel dysfunction compared to Non‐Type D personality. It is important to note, however, that participants in the Non‐Type D group did have slightly lower mean scores on the LARS scoring at each time point, indicating that they may have had slightly better bowel function overall.

## 4 DISCUSSION

To our knowledge, there have been few studies on whether certain personality traits are associated with LARS. The results of the study may offer new perspectives on the complex variables, such as personality traits, that affect functional outcomes following rectal cancer surgery. According to the univariate analysis of this study, there were significant positive correlations between LARS scores at each of the three‐time points, tumors in the lower rectum, and patients receiving neoadjuvant CCRT. The LARS score was positively correlated with Type A, Type D, and Type D‐SI personality traits 1 month after surgery. Furthermore, the multivariate analysis revealed a significant correlation between Type A personality and tumor level from the anal verge for the LARS score at 1 month following surgery. Additionally, tumor level from the anal verge and neoadjuvant CCRT was substantially associated with the LARS score at 6 months and 1 year after surgery, respectively.

### 4.1 Neoadjuvant CCRT with LARS syndrome

Neoadjuvant CCRT is a combination of chemotherapy and radiation therapy administered before surgery to shrink tumors and improve the chances of complete surgical removal.^31^, ^32^ However, neoadjuvant CCRT can also have side effects that may contribute to the development of LARS.^33^ For example, radiation therapy can damage the nerves and muscles in the pelvic region, resulting in bowel dysfunction that might present as diarrhea, constipation, or rectal bleeding.^34^ Chemotherapy can also cause changes in bowel function and may increase the risk of diarrhea, a common symptom of LARS.^35^ Battersby et al.^36^ predicted bowel dysfunction severity before anterior resection using the nomogram method with various preoperative covariates, including age, tumor height, total versus partial mesorectal excision, stoma, and preoperative radiotherapy. Another study by Emmertsen^25^ found that patients who received neoadjuvant CCRT had a higher risk of LARS than patients who did not receive neoadjuvant CCRT, particularly in patients with a higher BMI and those who had a longer delay between neoadjuvant CCRT and surgery. Multiple investigations have demonstrated the interaction between neoadjuvant CCRT and LARS in rectal cancer patients, suggesting that patient characteristics and the timing of neoadjuvant CCRT may be significant predictors of LARS.

Much about this subject still needs to be clarified because only some studies have examined the potential association between certain personality qualities and LARS, even though we assume there must be some linkage. In the univariate analysis of this study, rectal cancer patients with higher Type A and Type D personality scores were significantly associated with LARS. This suggests that personality characteristics may substantially influence the emergence of LARS. However, the exact mechanism underlying the relationship between personality types and LARS still needs to be fully understood, and further research is required to clarify this connection.

Some studies suggest that Type A personality traits, such as being competitive, ambitious, and driven, may contribute to increased stress and higher anxiety levels, which in turn may affect bowel function and increase the risk of LARS. Stress and anxiety can cause alterations in the gut–brain axis and affect the motility and sensitivity of the intestines,^37^ potentially leading to bowel dysfunction. Additionally, one study showed that patients with Type A personality structures had higher self‐esteem scores,^38^ which might lead to fewer adaptive coping strategies, such as less effective problem solving and seeking social support, which could further exacerbate the negative effects of stress on bowel function.

On the contrary, Type B personalities may be more resilient in coping with stress, including stress related to illness. They may be more likely to use positive coping strategies, such as seeking social support, engaging in physical activity, and maintaining a healthy lifestyle.

Alternatively, patients with Type D personalities tend to have more emotional stress, anxiety, and depression, which could make it more challenging to manage the pressure of surgery and recovery. Emotional distress has been related to elevated levels of cortisol, a stress hormone, that can trigger alterations in gastrointestinal motility and contribute to bowel dysfunction.^39^ In addition, Type D personality traits may result in less constructive coping strategies such as social withdrawal and rumination, which can exacerbate the detrimental effects of stress on bowel function. As a result of the emotional issues, they confront during surgery and rehabilitation, patients with Type D personalities may be at a higher risk of developing LARS. Healthcare professionals can play a critical role in recognizing patients with Type D personalities and offering them the proper emotional assistance and instruments to assist them in coping with the pressure of recovery from surgery.

Ultimately, it is crucial to determine which type of personality characteristic each patient exhibits and then offer the appropriate support approaches. Health practitioners can reinforce these coping strategies by providing patients with information about resources and activities that improve their physical and mental health. For instance, they may suggest support networks, exercise programs, and relaxation strategies to help patients manage their LARS.

Rectal cancer patients' personalities may benefit from nursing interventions that focus on stress management and coping strategies. These interventions may include cognitive–behavioral therapy,^40^ relaxation techniques, and mindfulness meditation.^41^ Some research indicates that anxiety and depressive symptoms positively correlate with physical, psychological, and social factors.^42^ Medical professionals might serve as a great source of information for patients developing positive coping strategies,^43^ including providing information on support groups that facilitate socially active communication, stress management techniques, and social support interventions.^38^

### 4.2 Clinical implications

In addition, healthcare providers can provide details concerning additional resources, such as counseling programs and online communities where patients can communicate with others experiencing similar situations. Patients with comparable symptoms could explore the connection between functional impairment and coping strategies following surgery for rectal cancer as well as nursing interventions to support these patients.

This study's multivariate analysis revealed that neither Type A nor Type D personality traits were associated with LARS after 6 months or 1 year, suggesting that the relationship between personality traits and LARS may decline over time. Further research may be necessary to determine whether it is feasible to alleviate the stress and depression symptoms of LARS as promptly as possible if medical professionals make individualized interventions based on the patient's characteristics. This work highlights the importance of further research employing larger sample numbers, multivariate analysis, and longitudinal designs to uncover potential causal relationships between personal characteristics and LARS.

When examining the temporal relationship between Type A and Type D personality qualities and LARS, we proposed that it is crucial to consider the dynamic nature of psychosocial factors and their potential influence on immediate outcomes. The primary stress responses, coping mechanisms, or affective states triggered by the surgical experience may influence the direct correlation. Type A individuals, characterized by their competitiveness and time urgency, may exhibit acute stress responses after surgery, which can affect short‐term LARS outcomes. We propose that rehabilitation standards and well‐defined action plans provide security for assertive, efficiency‐driven Type A personalities. Incorporating self‐management practices, such as goal‐setting and prioritizing a streamlined recovery process with attainable milestones, are crucial considerations. Besides, Type D individuals, characterized by a combination of negative affectivity and social inhibition, may experience heightened emotional responses immediately following the procedure. Our perspective is that Type D individuals, who are susceptible to experiencing emotional distress, could potentially gain advantages from an approach characterized by compassion and empathy. Establishing an open communication channel enables them to articulate their concerns while fostering or reinforcing social support networks, which become integral to their emotional well‐being.

These personality traits can influence early recovery, symptom perception, or the psychosomatic experience of LARS. However, over time, adaptation, coping mechanisms, and the normalization of the postsurgical experience may alleviate these initial associations, which explains the weakening of the correlation in the medium term. For better understanding, more research should be conducted regarding the specific ways that Type A and Type D traits affect short‐term LARS outcomes, focusing on psychological and physiological responses in the immediate postoperative period. This would help us better understand how personality traits interact with the evolving trajectory of LARS over time.

Patient education and counseling play a vital role in this context. Offering customized information about potential challenges in the short term, taking into account personality traits, empowers patients to cope effectively. Counseling sessions guide managing stress, setting expectations, and addressing emotional responses during the early stages of recovery. Integration into comprehensive care plans is another aspect of translating these findings into clinical practice. Personality assessments can be incorporated into holistic care plans for colorectal cancer patients undergoing surgery.

In summary, the clinical implications involve a shift toward individualized care that recognizes the role of personality traits in shaping the short‐term experience of LARS. By incorporating these findings into clinical practice, healthcare professionals can enhance patient outcomes and contribute to a more patient‐centered approach to colorectal cancer care.

### 4.3 Study limitations

Personality traits and LARS symptoms are self‐reported, which can lead to biases such as recall bias or social desirability bias. For instance, if a questionnaire is administered in a high‐pressure or stressful environment, it could influence how a respondent replies and skew the results. Moreover, social pressure or conformity bias may influence responses if the questionnaire is conducted in a group setting. To avoid this kind of bias as much as possible, we attempted to present the questionnaire in a neutral, nonthreatening environment and ensure that the instructions were clear and easy to follow for the patients in our study. Second, despite controlling for the basic clinicopathological characteristics, the study did not adjust for factors that might have influenced the outcomes, such as socioeconomic status and comorbidities. This study did not examine the socioeconomic variables because Taiwan's healthcare insurance framework is a nationwide obligatory participation framework, where all citizens are obliged to partake and contribute the corresponding healthcare insurance premiums. This premium framework upholds equity in sharing the burden, mitigating the financial hardship of economically disadvantaged groups. Taiwan's healthcare insurance framework offers broad coverage of medical services, comprising primary preventive care, diagnostic and treatment services, hospitalization, and surgeries. This makes it possible for everyone to access high‐quality medical care, regardless of their financial situation. Third, as far as we know, no specific verification study of the Bortner scale has been conducted in Taiwan. Further investigation is warranted to examine the suitability and dependability of this scale within the particular context of Taiwan, as indicated by the absence of empirical assessment. However, its practicality and reliability are somewhat questioned, considering its extensive adoption and recognition within Taiwanese society. In addressing the limitations of our study, it is crucial to acknowledge the constraint posed by the sample size. The limited number of participants may have curtailed the ability to detect statistically significant effects, emphasizing the need for caution in generalizing findings. While representative of our target population, the sample might only partially capture the diversity present. The challenge of establishing causal relationships is inherent, given the observational nature of our study. To make future investigations more robust, it is recommended to increase the sample size to improve the statistical power. Moreover, it may be useful to use a longitudinal research design to gain a more detailed understanding of the dynamic relationships between variables over time. We suggest that future studies use more rigorous methodologies, such as experimental designs or better control of additional variables in observational studies, to enhance the understanding of the research question.

### 4.4 Conclusions

This study sheds light on the potential correlation between specific personality traits and the occurrence of LARS in rectal cancer patients. In this investigation, we have brought attention to the association between LARS and psychosocial functioning, emphasizing the potential influence of personality characteristics on coping after illness. While the causal relationship remains ambiguous, healthcare practitioners could employ this information to identify patients more susceptible to developing LARS and offer appropriate interventions, such as social support programs and stress management strategies.

Additional research is necessary to completely comprehend the underlying mechanisms underlying the association between personality types and LARS and to assess the efficacy of nursing interventions emphasizing stress management and coping mechanisms.

## AUTHOR CONTRIBUTIONS

**Jeng‐Fu You:** Conceptualization (lead); data curation (equal); formal analysis (equal); funding acquisition (equal); investigation (lead); methodology (lead); project administration (equal); resources (equal); software (lead); supervision (equal); validation (equal); visualization (equal); writing – review and editing (equal). **Ting‐Yu Chiang:** Conceptualization (equal); data curation (equal); formal analysis (equal); funding acquisition (equal); investigation (equal); methodology (equal); project administration (equal); resources (equal); software (equal); writing – original draft (equal). **Yu‐Jen Hsu:** Data curation (supporting); writing – review and editing (supporting). **Yih‐Jong Chern:** Data curation (supporting); writing – review and editing (supporting). **Chun‐Kai Liao:** Data curation (supporting); writing – review and editing (supporting). **Wen‐Sy Tsai:** Validation (supporting). **Pao‐Shiu Hsieh:** Validation (supporting). **Hung‐Chih Hsu:** Data curation (supporting); validation (supporting). **Hsiu‐Lan Lee:** Validation (supporting). **Yu‐Fen Lin:** Validation (supporting).

## FUNDING INFORMATION

Chang Gung Memorial Hospital, CMRPG3K1991. This study was supported by a grant from the Chang Gung Memorial Hospital (CMRPG3K1991) Research Program in Taiwan.

## CONFLICT OF INTEREST STATEMENT

The authors have no competing interests to declare that are relevant to the content of this article.

## ETHICS STATEMENT

This study was performed in line with the principles of the Declaration of Helsinki. Approval was granted by the Ethics Committee of Chang Gung Medical Foundation in Taiwan, and a permission certificate was obtained (Number: 202000669B0).

## CONSENT TO PARTICIPATE

Informed consent was obtained from all individual participants included in the study.

## CONSENT TO PUBLISH

The authors affirm that human research participants provided informed consent for publication.

## Data Availability

Data are available on request due to restrictions. The data presented in this study are available on request from the corresponding author. The data are not publicly available due to privacy.
